# Missing-Sheds Granularity Estimation of Glass Insulators Using Deep Neural Networks Based on Optical Imaging

**DOI:** 10.3390/s22051737

**Published:** 2022-02-23

**Authors:** Wenxiang Chen, Yingna Li, Zhengang Zhao

**Affiliations:** 1 Faculty of Information Engineering and Automation, Kunming University of Science and Technology, Kunming 650500, China; wenxiang.chen@stu.kust.edu.cn (W.C.); zhaozhengang@stu.kust.edu.cn (Z.Z.); 2 Computer Technology Application Key Lab of the Yunnan Province, Kunming 650500, China

**Keywords:** power transmission lines, insulators, missing sheds, deep neural networks, attention mechanism

## Abstract

Insulator defect detection is an important task in inspecting overhead transmission lines. However, the surrounding environment is complex, and the detection accuracy of traditional image processing algorithms is low. Therefore, insulator defect detection is still mainly performed manually. In order to improve this situation, we proposed an insulator defect detection method called INSU-YOLO based on deep neural networks. Overexposure points in the image will interfere with insulator detection, so we used image augment to reduce noise and extract the edge information of the insulator. Based on an attention mechanism, we introduced a structure called attention-block where the backbone extracts the feature map, and this aims to improve the ability of our method to detect insulators. Insulators have a variety of specifications, and the location and granularity of defects are also different. Therefore, we proposed an adaptive threat estimation method based on the area ratio between the entire insulator and the defect area. In addition, in order to solve the problem of data shortage, we established a dataset called InsuDetSet for model training. Experiments on the InsuDetSet dataset demonstrated that our model outperforms existing state-of-the-art models regarding both the detection box and speed.

## 1. Introduction

The main function of insulators is electrical insulation and line support, which are critical for power transmission. Large overhead transmission lines are one of the most important methods of power transmission and are mainly distributed in places with complex environments, such as mountains, forests, and suburbs. High-voltage overhead transmission line insulators are mainly divided into the following three types: ceramic insulators, composite insulators, and glass insulators. Affected by weather, human activities, etc., insulators are prone to defects, such as contamination, icing, cracks and missing sheds. Among them, missing sheds are one of the most prone and threatening defects because they will directly affect the insulation and mechanical support performance of the insulator. Electric breakdown, wind, and sun often cause insulators to lose sheds, which is fatal to the performance of the entire insulator string. Therefore, detecting missing sheds is imperative, but the surrounding environment of overhead transmission lines complicates this detection task [1,2].

Because of the low cost and simple use of optical image sensors, there have been many detection methods based on image and visual technology in recent years. Traditional image processing algorithms have a fast computation speed and low resource consumption. However, it is easy to obtain a lower detection accuracy when dealing with insulator defect areas, because similar information will interfere with them in the background [3,4].

The method based on a deep neural network is the most accurate one at present and has also been the main research direction in recent years [5,6]. Strong feature extraction ability, the ability to characterize latent information, and a large amount of training data are key to the excellent performance of this method. The challenge of deep neural networks is achieving balance between detection speed and resource consumption.

Detection methods based on additional equipment are also limited by the complexity and service life of the operating machinery [7,8,9]. Stability in the power system is necessary to protect human activities. Therefore, current defect detection of insulators of overhead transmission lines is mainly carried out manually.

**Figure 1:**
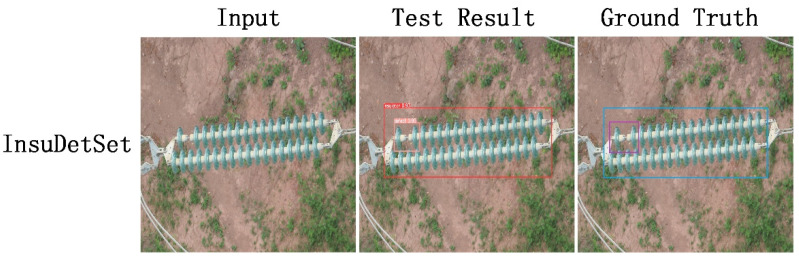
Test examples of our model on InsuDetSet.

In view of the current research status of insulator defect detection, as shown in Figure 1, we propose an analysis method of insulator defect detection and threat level based on a deep neural network. The main contributions of this paper are as follows:

- Based on the YOLOv4 framework, we propose an insulator detection model called INSU-YOLO, which is robust compared to traditional detection algorithms. In addition, we produced an insulator detection dataset called InsuDetSet based on real images.
- We use Gaussian blur to smooth the overexposed points in the image and use the canny operator to extract edge information to enhance the semantic information in the image, which helps the model to learn the insulator region.
- We propose a structure called attention-block, based on the attention mechanism. This method strengthens the feature map extraction ability of our model and effectively improves detection accuracy.
- In order to estimate the degree of the missing-shed phenomenon in insulators with different specifications, we propose a threat level estimation method based on the area of the defect area.

The remainder of this paper is organized as follows. Section 2 briefly introduces related work in insulator defect detection. Section 3 introduces the basic framework used in the method proposed in this article. In Section 4, we introduce the details of the use of image enhancement technology and introduce the proposed attention-block and threat level estimation methods. In Section 5, we introduce the dataset, the experimental details, and a series of comparative experiments. Section 6 provides a brief summary of the work of this paper.

## 2. Related Work

Existing image-based research about insulator defect detection is mainly divided into two categories: traditional methods and methods based on deep neural networks.

Traditional insulator defect detection methods usually combine image processing and machine learning techniques. Huang et al. [10] proposed K-means clustering analysis based on the red-blue difference and the weighted gray value to extract defective insulators from the image, but this is prone to calculation deviations when the light is strong. Yin et al. [6] proposed an algorithm, called the double parity morphological gradient, to extract insulator strings, but the algorithm is based on infrared images. Iruansi et al. [11] used a combination of the active contour method and morphological operation to segment insulators and water droplets; the detection effect of this method was obtained by comparison with the true value of the artificially created target. Yu et al. [12] proposed an active contour model algorithm based on the insulator’s texture and shape and defined a new energy function based on the extracted features. Generally, this kind of method has a faster operation speed, but it is highly dependent on the image quality and the robustness of the extracted features.

Since AlexNet [13] was proposed in 2012, deep neural networks, especially convolutional neural networks, have been the hottest research direction in the field of vision. Classical object detectors are usually divided into two parts, a pre-trained feature extraction backbone, such as VGG [14], ResNeXt [15] and DenseNet [16], and a head for detecting object categories and locations, such as R-CNN [17], Fast R-CNN [18], Faster R-CNN [19], R-FCN [20] and Libra R-CNN [21]. There is also a certain amount of research on one-stage object detection, such as SSD [22], RetinaNet [23], and YOLO [24,25,26]. In recent years, some anchor-free class methods, such as CornerNet [27,28], FCOS [29], CenterNet [30] have also appeared.

At present, research in insulator detection and defect recognition based on deep neural network mainly employs existing, state-of-the-art methods. Sadykova et al. [31] used the YOLOv3 [26] neural network model to train a classifier to detect insulators and interference objects to solve the problem of ice, water, and snow attached to insulators, and this model could accurately determine the location of insulators with real-time data. Zhao et al. [32] analyzed and adjusted the anchor point generation method and non-maximum suppression in Faster R-CNN according to the different sizes, aspect ratios, and mutual occlusion of the insulators in the image. Liu et al. [33] built an insulator detection method based on YOLOv3 and Dense-Blocks and combined it with a multi-level feature mapping module for different sizes of insulators and complex aerial images. Chen et al. [34] proposed a detection method for foreign objects attached to lines, based on Mask R-CNN. Kang et al. [35] proposed a surface defect detection system, based on Faster R-CNN, for defective insulators in high-speed rail networks and achieved the classification of insulators and abnormal types through classifiers and noise reduction autoencoders. In order to improve the adaptability of insulator detection, Liu et al. [36] proposed a defect classification automatic encoder based on unsupervised learning; the defect level is judged by the density-based spatial clustering index with noise. Xiao et al. [37] used the difference in calorific value between normal and faulty areas of insulators, introduced the K-means clustering method to eliminate bad data, and proposed a fault detection method for insulator strings based on infrared image analysis and a probabilistic neural network. However, this method is susceptible to interference from abnormal weather and temperature. For the internal defects of insulators, Cheng et al. [38] used optimized infrared detection methods to reduce the influence of humidity on the detection, but the operation of removing the influence of humidity may cause the faulty insulator to be falsely detected as a normal insulator. In order to detect failures in components of power lines along a railway, J. P. Augusto Costa and O. A. Carmona [39] achieved high-precision detection results based on Tiny Yolo in simulating a real-world laboratory to meet the requirements of real-time detection.

In addition, there are methods for defect detection based on the physical characteristics of insulators using additional mechanical instruments, such as density analysis [7], laser instruments [8], and ultrasonic measurement methods [9]. Some researchers use infrared radiation inspection [40] to detect the heating state of the insulator. However, these methods are limited by the life of the instrument and are not convenient for use on overhead power lines.

It can be inferred from the above research that the current insulator identification and defect detection methods are generally limited by a complex background and by the image quality, and the detection accuracy needs to be improved. Our summary of these methods is as follows:

- Traditional image processing algorithms have a fast calculation speed and low resource requirements and are suitable for real-time operation on edge devices. However, this kind of method relies heavily on the completeness of the feature extractor, has high requirements on the quality of the input image, and is susceptible to interference from strong light and the background. At present, the detection accuracy of these algorithms is weaker than that of deep neural network algorithms; it is mainly suitable for situations with a simple background, small environmental changes, and high real-time requirements.
- The insulator defect detection algorithm based on deep neural networks has excellent accuracy, but the current research mainly applies state-of-the-art methods, and there are few studies on detailed analysis of the defect area. In addition, many studies are based on infrared images, which require additional infrared instruments. Moreover, most of the methods with outstanding results have high time complexity. We hope that the proposed method can run in real time on edge devices, such as drones.
- In addition, some researchers use the physical characteristics of insulators for defect detection. This type of method usually requires additional equipment costs and is not friendly to already installed insulators.

In view of the above research, we hope to provide a method with low resource consumption, fast calculation speed, and high detection accuracy based on vision technology. Therefore, we focused on a one-stage target detection method within the deep neural network, combined with the actual background of the data and the visual characteristics of the insulator, and proposed an insulator defect detection method.

## 3. Basic Components of INSU-YOLO

The background of the line inspection image is complicated, and it is difficult to construct the structure and color characteristics of the insulator. Therefore, we decided to perform the task of detecting and defect location of the glass insulator based on a deep neural network. A deep neural network is an end-to-end method that can add highly abstract guidance information to the detection task. Based on ResNet101 [41] and YOLOv4 [42], we built a one-stage neural network algorithm called INSU-YOLO, which can achieve a balance between running speed and detection accuracy.

Redmon et al. proposed the YOLO [24] model in 2016. The YOLO algorithm divides the input image into S*S grids and calculates the class of each grid that falls into the object. It is also responsible for the prediction of this type of object during the test. The YOLO model has been continuously improved in recent years, and the most representative version is YOLOv4 proposed by Alexey et al. Because of the PAN structure [43] and the use of a large number of training tricks, the detection accuracy of YOLOv4 has been greatly improved. As shown in Equation (1), the loss function of YOLOv4 consists of three parts:

(1) $$L_{det}=L_{box}+L_{obj}+L_{cls}$$

where $L_{box}$, $L_{obj}$, and $L_{cls}$ represent the regression loss, confidence loss, and category loss of the box, respectively. The expression of the box regression loss is as follows:

(2) $$L_{box}=\lambda_{coord}\sum_{i=0}^{S^{2}}\sum_{j=0}^{B}1_{i,j}^{obj}\left(1-\left(\mathrm{IoU}-\frac{Distance\_2^{2}}{Distance\_C^{2}}-\frac{v^{2}}{\left(1-\mathrm{IoU}\right)+v}\right)\right)$$

where $\lambda_{coord}$ is the weight of box regression loss, $S_{i}^{2}$ represents the *i*th grid of S*S size, $B_{j}$ represents the *j*th predicted box of $S_{i}^{2}$, and $1_{i,j}^{obj}$ indicates that there is a target center of the prediction category in the box. IoU is the Intersection-of-Union of the predicted box and ground truth, the calculation formula of IoU is Equation (3), Distance_2 is the Euclidean distance between the center coordinates of $Box^{p}$ and $Box^{gt}$, Distance_C is the diagonal length of the smallest bounding rectangle of $Box^{p}$ and $Box^{gt}$, v is a parameter to measure the consistency of the aspect ratio of $Box^{p}$ and $Box^{gt}$, and the calculation formula of v is Equation (4).

(3) $$\mathrm{IoU}=\frac{\left|Box^{p}\cap Box^{gt}\right|}{\left|Box^{p}\cup Box^{gt}\right|}$$

where $Box^{p}$ and $Box^{gt}$ represent the predicted box and ground truth, respectively.

(4) $$v=\frac{4}{\pi^{2}}{\left(arctan\frac{w^{gt}}{h^{gt}}-arctan\frac{w^{p}}{h^{p}}\right)}^{2}$$

where $w^{gt}$ and $w^{p}$ represent the width of the ground truth and predicted box, respectively, and $h^{gt}$ and $h^{p}$ represent their respective heights.

Similar to the regression loss, the loss function for the target prediction confidence is as follows:

(5) $$L_{obj}=\lambda_{noobj}\sum_{i=0}^{S^{2}}\sum_{j=0}^{B}1_{i,j}^{noobj}{\left(c_{i}-{\hat{c}}_{i}\right)}^{2}+\lambda_{obj}\sum_{i=0}^{S^{2}}\sum_{j=0}^{B}1_{i,j}^{obj}{\left(c_{i}-{\hat{c}}_{i}\right)}^{2}$$

where $\lambda_{noobj}$ and $\lambda_{obj}$, respectively, represent the weight of the confidence loss when the object is not included and when it is included. $c_{i}$ and ${\hat{c}}_{i}$, respectively, represent the true value and predicted value of whether there is an object of category *i* in the current box. The other parameters have the same meaning as in the regression loss.

The category prediction loss uses the classic cross-entropy loss, and its calculation formula is as follows:

(6) $$L_{obj}=\lambda_{noobj}\sum_{i=0}^{S^{2}}\sum_{j=0}^{B}1_{i,j}^{noobj}{\left(c_{i}-{\hat{c}}_{i}\right)}^{2}+\lambda_{obj}\sum_{i=0}^{S^{2}}\sum_{j=0}^{B}1_{i,j}^{obj}{\left(c_{i}-{\hat{c}}_{i}\right)}^{2}$$

where $\lambda_{class}$ represents the weight of the category loss, ${\hat{p}}_{i}(c)$ represents the predicted value of the confidence of the current category, $p_{i}(c)$ is a conditional probability, which is obtained by obtaining a value of 0 or 1, depending on whether $S_{i}^{2}$ contains the target center, and then multiplying it with IoU.

YOLOv4 uses CSPDarknet53 [44] as the feature extraction network, but CSPDarknet53 has a large number of parameters. In addition, the only object to be detected in this paper is the insulator. As shown in Table 1, ResNet101 is composed of multiple groups of residual blocks. ResNet has an excellent effect of extracting feature maps, which overcomes the problem of low learning efficiency caused by excessive network depth. Therefore, we decided to use the classic ResNet101 as our backbone.

**Figure 2:**
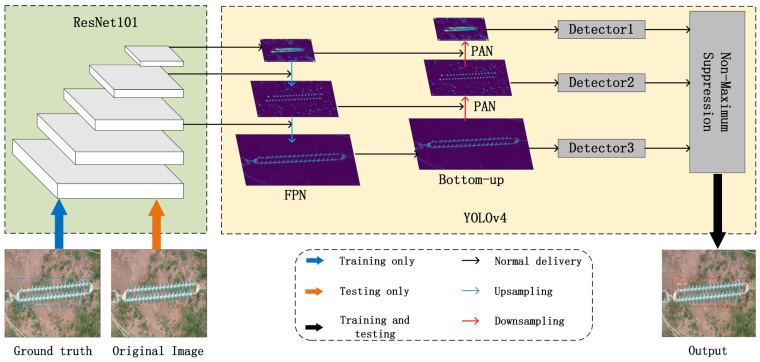
The basic framework of the INSU-YOLO containing two parts: the ResNet101 module and the YOLOv4 module. First, ResNet101 is used to obtain feature maps of different levels of abstraction; FPN and bottom-up structures using PAN in YOLOv4 are then used for feature transfer; finally, we use the detector for classification and box regression to obtain the output image.

As shown in Figure 2, we used ResNet101 and YOLOv4 to build our insulator detection model. The input image first obtains multiple feature maps through ResNet101, and YOLOv4 builds the FPN [45] network based on the feature maps of the 3rd, 4th, and 5th layers. The feature map then goes through a bottom-up structure and uses PAN [43] to associate with FPN. The advantage of this approach is that it can improve the abstraction level of the feature map while preserving the lowest level features. Finally, multiple detectors are used to detect insulators, and non-maximum suppression [46] is used to screen the detection results.

## 4. Insulator Detection Using INSU-YOLO

**Figure 3:**
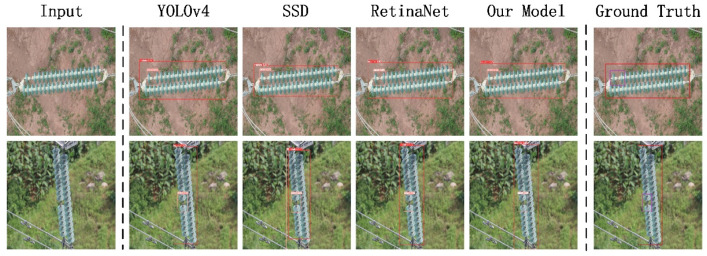
Test examples of one-stage class detectors on InsuDetSet, including our benchmark model YOLOv4, classic SSD and RetinaNet, and our model. The results show that our model outperforms other one-stage models.

The results in Figure 3 show that the combination of ResNet101 and YOLOv4 achieved good results, but there is still room for improvement. Therefore, based on image processing technology and the attention mechanism [47], we have carried out more targeted work on image noise and semantic information, making INSU-YOLO more suitable for the task of insulator defect detection. Specific details will be introduced in this section.

### 4.1. Image Augment

Image quality is critical for improving the detection accuracy of the model, but the reflection of light causes a large number of overexposed points in the image, so we needed to remove this noise first. In addition, we used edge detection algorithms to highlight the contour information of objects in the image. The image has been enhanced by the above two methods.

#### 4.1.1. Image Denoising

The inspection image of the transmission line was obtained by a drone, and the image was susceptible to the influence of strong light and produced overexposure points during imaging. Our first step was then to remove the noise, because the noise area changes greatly, and it easily interferes with the insulator detection.

We used a Gaussian filter [48] to smooth the image noise. Compared with a mean filter [49] and median filtering [50], a Gaussian filter assigns different weights to pixels in different positions in the neighborhood, which can complete the denoising while retaining the overall gray-scale distribution characteristics of the image. To implement Gaussian filtering on the image, a (2*k* + 1) × (2*k* + 1) Gaussian filtering kernel is generally used to convolve the image. The kernel generation equation is given by the following formula:

(7) $$H_{ij}=\frac{1}{2\pi \sigma^{2}}\exp\left(-\frac{{\left(i-\left(k+1\right)\right)}^{2}+{\left(j-\left(k+1\right)\right)}^{2}}{2\sigma^{2}}\right);1\leq i,j\leq \left(2k+1\right)$$

where *k* represents an integer, (2*k* + 1) represents the size of the convolution kernel, and (*i*, *j*) represents the coordinates of one of the points.

**Figure 4:**
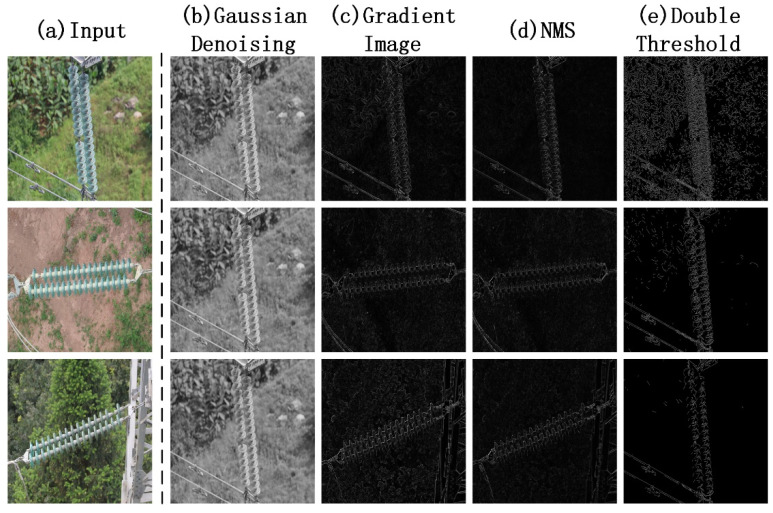
Test examples of image augment. (**a**) The original input, (**b**) the Gaussian denoising comparison of the same image, (**c**) the gradient image, (**d**) the image after NMS processing, and (**e**) the edge image obtained by the double-threshold segmentation method.

The size of the convolution kernel is usually an odd number. Generally speaking, the larger the size of the convolution kernel, the stronger the denoising ability. In order to obtain the most suitable convolution kernel size, we selected a kernel size of (2*i* + 1) × (2*i* + 1) (*i* = 1, 2, 3) for experimental comparison. Figure 4b shows the effect of 3 × 3, 5 × 5, and 9 × 9 convolution filters on image denoising. The image processed by the 3 × 3 convolution kernel still has a high amount of noise, and the 9 × 9 kernel has excessive denoising and lost part of the insulator shed; the 5 × 5 convolution kernel has the best processing effect.

In addition, although there is still a certain amount of noise in the background, this is not a problem because the network will focus on the insulator information in the label during training. A Gaussian filter reduces the noise of the input image, but it also leads to blurred image boundaries, which will affect the positioning task of the insulator. Thus, in the next section, we focus on improving the semantic information in the image.

#### 4.1.2. Edge Extraction

The side effect of a Gaussian filter is that the edges of the image become blurred. We decided to perform a contour extraction operation on the image to enrich the semantic information of the image.

Edge extraction requires that the gradient of the image is calculated first. The gradient can reflect the change of the pixels in the area. The greater the change in the gradient, the greater the difference between the pixels in the area. When zooming in on the entire picture, the area with edges usually changes sharply. Gradient calculation is divided into size and direction. The commonly used image gradient calculation formulas in the horizontal and vertical directions are as follows:

(8) $$\frac{\partial f}{\partial x}\approx \frac{f(x+1,y)-f(x-1,y)}{2}$$

(9) $$\frac{\partial f}{\partial y}\approx \frac{f(x,y+1)-f(x,y-1)}{2}$$

The gradient direction θ and increase $\left\|\nabla f\right\|$ of the pixel can then be calculated, and the calculation formula is as follows:

(10) $$\theta =\tan^{-1}(\frac{\partial f}{\partial y}/\frac{\partial f}{\partial x})$$

(11) $$\left\|\nabla f\right\|\;=\;\sqrt{{\left(\frac{\partial f}{\partial x}\right)}^{2}+{\left(\frac{\partial f}{\partial y}\right)}^{2}}$$

Through the above calculation, as shown in Figure 4c, we obtain the image gradient. The place where the gray level changes may or may not be an edge, and we obtain a collection of all pixels that may be edges. However, these edges are too thick, and the original one edge is replaced with several overlapping edges, which makes it look thick visually.

Therefore, we decided to use non-maximum suppression [48] to reduce these edges, retain the largest grayscale changes in the horizontal, vertical, and diagonal directions within the eight neighborhoods of the pixels, and eliminate other pixels. The wide edge composed of pixels became a single-pixel-wide edge. The processing result is shown in Figure 4d.

After the non-maximum suppression, there were still a certain number of noise points in the image, and we used the dual threshold method [48] to filter them. That is, we set an upper threshold and a lower threshold. If a pixel in the image was greater than the upper threshold, it was considered as a boundary (strong edge), and if it was less than the lower threshold, it was not a boundary. Pixels between these two were considered candidates (a weak edge).

The threshold setting of a strong edge is very important. Its value setting is generally high so that the gradient value of the pixel is required to be large enough, and the area of the image changes sharply. In order to obtain a suitable threshold for the previous setting, we selected multiple values for sensitivity analysis. The lower bound is generally 0.5 times the upper bound [48]. Figure 4e shows the effect of the previous session of 200, 300, and 400. It can be seen that the effect is best when the upper bound is 300. The weak edge may be an edge or noise. When there are strong edge points in the surrounding eight neighborhoods of the weak edge, the weak edge point is turned into a strong edge point, so as to supplement the strong edge. The pixel classification calculation is expressed as follows:

(12) $$f\left(i\right)\;=\;\left\{\begin{array}{c} \mathrm{strong}\;\mathrm{edge}\;;\;\;\;\;i>300\\ \mathrm{weak}\;\mathrm{edge}\;;\;150\leq i\leq 300\\ \mathrm{non}-\mathrm{edge}\;;\;\;\;\;i<150 \end{array}\right.$$

### 4.2. Attention Mechanism

The quality of the input image was improved after image augmentation. We hope to introduce the positive effects of these color and semantic changes into network training. Therefore, based on the attention mechanism, we propose a feature enhancement block called attention-block to improve the extraction effect of feature maps.

Attention-block first needs to perform a convolution operation on the input image and edge image to obtain the attention weight matrix ${\left\{I_{A}^{i}\right\}}_{i=1}^{2}$ corresponding to the image. The calculation process is as follows:

(13) $$I_{A}^{i}=\mathrm{Softmax}(I^{i}W_{A}^{i}+b_{A}^{i}),\mathrm{for}\;i=1,2$$

where $I^{i}$ represents the input image, ${\left\{W_{A}^{i},b_{A}^{i}\right\}}_{i=1}^{2}$ represents the parameter of the convolution operation, and $\mathrm{Softmax}\left(\cdot \right)$ represents the SoftMax function used for normalization. We multiplied the resulting attention weight matrix with the corresponding input image to obtain the final output:

(14) $$I_{A}=(I_{A}^{1}⊗I^{1})⊕(I_{A}^{2}⊗I^{2})$$

where $I_{A}$ represents the final output result of the attention mechanism, $I^{1}$ and $I^{2}$ represent the input images, and the symbols ⊗ and ⊕ represent the multiplication and addition elements of the matrix.

**Figure 5:**
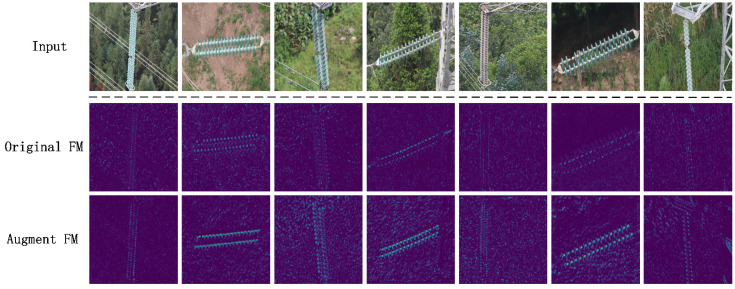
Input represents the original input image, Original FM represents the feature map of the third layer output of the original ResNet101, and Augment FM represents the output of the third layer of ResNet101 after being enhanced by the attention mechanism. The features in the Augment FM output picture are more obvious, which is more conducive to the convergence of the model.

We applied attention-block to the first, second, and third layers of ResNet101. The abstraction level of the fourth- and fifth-layer feature maps was higher, so attention-block was no longer used. As shown in Figure 5, the feature map extracted after adding the attention-block has clearer and more obvious features than the feature map extracted from the original image only. After all framework components were introduced, we trained INSU-YOLO. The training process is as described in Algorithm 1.
**Algorithm 1.** The training process of INSU-YOLO.Input: Original insulator image set $I=\left\{I_{1},\cdot \cdot \cdot ,I_{N}\right\}$ that each image contains insulators, and some images contain defective insulators.Output: INSU-YOLO after training.1: Initialize INSU-YOLO with random weights;2: repeat3:   for *i* in 1~epochs do4:     for *j* in 1~N do5:       Image augment for $I_{j}$; 6:       Extract feature map using ResNet101;7:       Output detection results using YOLOv4;8:       Calculate the penalty value via Equation (2),(5) and (6);9:       Minimize Formula (1) to update the parameters of INSU-YOLO;10:     end for11: end for12: until INSU-YOLO completes convergence13: return

### 4.3. Defect Granularity Estimation

There are different specifications of glass insulators in overhead transmission lines, and the granularity of missing sheds is also different. In response to this phenomenon, we propose an adaptive threat level estimation method based on the defect and overall area ratio. In practical application scenarios, it is usually not necessary to calculate the defect particle size of the insulator in the full frequency range. We only need to obtain the level of the defect and take corresponding measures according to it.

Table 2 shows the three missing-shed levels determined by the actual application scenarios and experimental calculations based on the ratio of the defect area to the entire insulator area. After obtaining the detection frame of the insulator and the defect area based on INSU-YOLO, we can calculate the area ratio of the two. Referring to the method of coal granularity estimation in [51], the defect granularity level to which the ratio size belongs can be found in Table 2. In fact, most glass insulators do not fail, so the ratio of the two is also 0. In addition, when the number of missing pieces of glass insulator strings is higher than 1, it is already a high threat, so the threshold of high-risk insulators is not too large.

**Figure 6:**
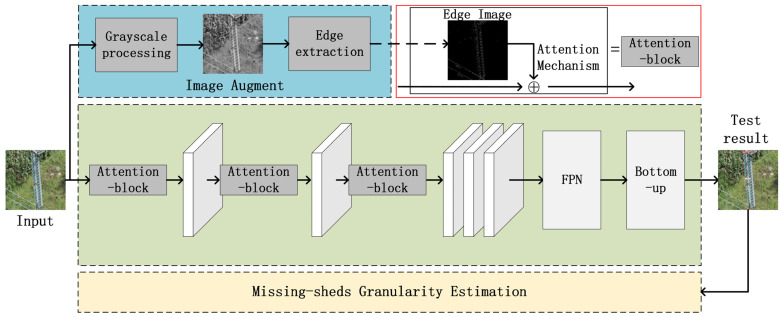
The realization of missing-shed granularity estimation of insulators is divided into three parts: image augment, INSU-YOLO, and missing-sheds granularity estimation, corresponding to image preprocessing, insulator detection, and defect analysis, respectively. First, image augment is used to improve the quality of the input image and provide edge images. We then use INSU-YOLO to obtain detection frames for insulators and defects. Finally, missing-sheds granularity estimation is used to analyze the degree of defects.

As shown in Figure 6, we used the image augment module, the INSU-YOLO framework, and the model for the estimation of missing-shed granularity to construct the entire insulator missing-sheds estimation process.

## 5. Experiments and Analysis

### 5.1. Experiment Description

#### 5.1.1. Dataset

An insulator dataset is necessary for model evaluation. The only publicly available dataset for insulator detection is CPLID [1]. Most of the data in this dataset are obtained through geometric transformations such as cropping and rotation, and are spliced with the background, which is not conducive to the application of the model in actual scenes. Therefore, we took the image of the glass insulators on a 500 KV overhead transmission tower line in a certain area of China based on an image sensor carried by a drone. The dataset was constructed using the acquired image data, in order to verify the effectiveness of our proposed model.

We used LabelMe as a marking tool to label all the insulators in the image. It should be noted that, when we marked the defect area, we reserved a part of the normal area. Due to the loss of shed, most of the defect area is background information, which makes the defect area highly susceptible to interference from the background information during training. The method of adding a part of normal sheds when marking the defect area can ensure difference from the background and help improve the detection accuracy of the model.

We named this dataset InsuDetSet, which consists of 3000 images. Approximately 10% of images contain defects of varying degrees. We randomly divided the experimental database into two subsets. The training database (TR-DB) contains 2500 images, and the test database (T-DB) contains 500 images.

#### 5.1.2. Experiment Configuration

The experiments used a personal computer with a hardware configuration of AMD R5-3600X CPU, 32GB RAM and NVIDIA RTX 2080 GPU. The system was Ubuntu 18.04, and the software environment was Pytorch1.4. All test programs were written based on the Python language. In terms of experimental hyperparameters, in general, the larger the training batch size setting, the better the model performance. Therefore, we set the batch size to 16 and iterated 200 epochs in total. The initial learning rate was set to 0.0001, the first 100 epochs remain unchanged, and the last 100 epochs gradually decrease to 0.

### 5.2. The Baselines

In the following experiments, we choose one-stage, two-stage and anchor-free methods as comparison methods.

YOLOv4 [42]: This method is the latest achievement of the YOLO series. After continuing the advantages of the previous work, it introduces the structure of FPN+PAN, which improves the transferability of features in the network, and is also the basis of our proposed model.

Cascade R-CNN [52]: This framework is the latest achievement of the R-CNN series. It creatively introduces a cascade structure. The detection accuracy is state-of-the-art, but its excellent performance consumes a lot of training resources. 

CenterNet [30]: This method is a heatmap-based detection method, not anchor-based, which has the advantage of fast testing and low space occupancy.

SSD [22]: SSD is also one of the classic one-stage object detection methods. It initially utilizes multiple detectors.

RetinaNet [23]: RetinaNet is based on FPN [45], and its contribution is to propose focal loss to solve the problem of category imbalance.

### 5.3. Qualitative Evaluation

**Figure 7:**
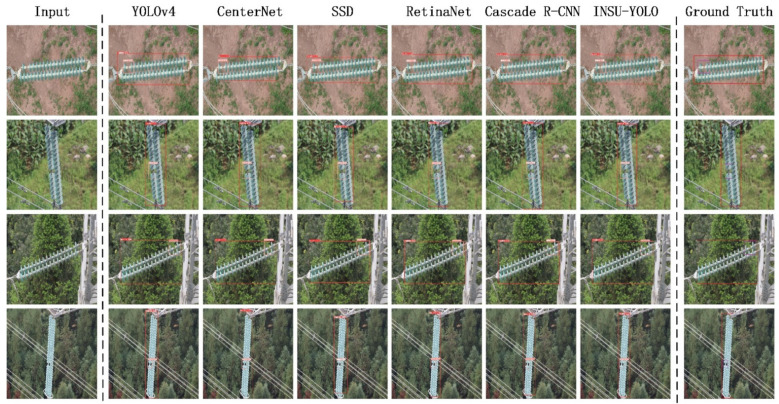
Test examples of each model on the InsuDetSet dataset. Experimental results show that the performance of INSU-YOLO is similar to Cascade R-CNN, better than SSD, RetinaNet and YOLOv4 in one-stage class, and CenterNet based on heatmap.

The qualitative experimental results are shown in Figure 7. The resolution of the test images was 608 × 608. The test results output by INSU-YOLO proposed in this paper were more accurate in category and location. When using the InsuDetSet dataset for testing, the detection results output by SSD were not accurate enough, and those of RetinaNet and YOLOv4 were better. Our INSU-YOLO is the same as the two-stage algorithm Cascade R-CNN. The output box is the most accurate compared to the ground truth, and INSU-YOLO consumes fewer resources than Cascade R-CNN. The results output by CenterNet are general, and we think this is because the scene of the insulator detection is too complicated.

### 5.4. Quantitative Evaluation

We used AP50–AP90 to evaluate the accuracy of the box position in testing. After that, we used missing-sheds granularity estimation to count the detection results of the baselines and compared them with the ground truth.

#### 5.4.1. Precision of Box

When evaluating the performance of all methods in the experiments, we used AP in the COCO dataset [53] as the evaluation criterion, which is based on the prediction results and the ground truth IoU. The calculation formula is shown in Equation (3), and the threshold of IoU was set to 0.5, 0.7, etc., respectively, to test the test performance of the model under different IoU thresholds. This approach can better evaluate the performance of the model.

As shown in Table 3, although the accuracy of INSU-YOLO showed a slight improvement compared to Cascade R-CNN, it was much higher than that of SSD and RetinaNet, which are also one-stage detectors. Compared with YOLOv4, the source of our model also showed a certain degree of improvement, which implies that the image augment and the attention mechanism play an important role. The experimental results showed that INSU-YOLO can effectively locate the position of the insulator in the image to achieve the purpose of defect detection. Furthermore, when the IoU threshold was 0.9, the detection accuracy was significantly higher than other baselines, which is one of the strengths of our model.

We also considered the test speed. In terms of FPS, Cascade R-CNN takes the longest time, INSU-YOLO is similar to other one-stage detectors, and CenterNet takes the least time. In general, the one-stage algorithm has the best balance between speed and accuracy, and the test speed of about 76 frames per second is sufficient to support real-time requirements.

#### 5.4.2. Defect Detection

In order to verify the effectiveness of the missing-sheds estimation proposed in this paper for defect detection, the results of INSU-YOLO and other baselines on the test set were calculated as shown in Table 2. As shown in Table 4, compared with the ground truth, our model was the most accurate for the statistics of normal insulators, small defect insulators, and large defect insulators. There was still a 1% omission, but we think this is allowable. The performance of Cascade R-CNN was also very high, and we believe that two-stage algorithms are still robust. CenterNet’s performance was average, but its performance in detection speed was sufficient. SSD, RetinaNet, and YOLOv4 performed slightly worse than Cascade R-CNN, but considering the test speed, the one-stage method was the most balanced in terms of speed and accuracy.

### 5.5. Sensitivity Analysis

In this section, we review multiple sets of sensitivity analysis performed on each component of INSU-YOLO, which includes the choice of backbone, image augment, the attention mechanism, the number of training iterations, and the minimum amount of training data.

#### 5.5.1. Backbone

We conducted a sensitivity analysis on the backbone used by INSU-YOLO while retaining other improvements. As shown in Table 5, the CSPDarknet53 used by YOLOv4 was improved based on ResNet50, so it performed better. However, we believe that its FPS can be further improved. In addition, the only objects we need to detect are insulators. Therefore, we believe that it may be more effective to expand the number of network layers and improve the feature abstraction ability of the backbone. The performance of ResNet101 also supports our idea, but if network layers, such as using ResNet152, continue to be added, the improvement is limited, so we decided to use ResNet101 as the backbone.

#### 5.5.2. Image Augment

To verify the effectiveness of preprocessing, we performed a sensitivity analysis on the image denoising and edge detection used, while keeping other improvements constant. Table 6 shows that, compared with not using any preprocessing strategy, using image denoising and edge extraction alone led to a certain improvement in detection effect. If both are used, the AP50 will increase by about 5 percentage points, which shows that the image augment method we use in this paper is effective.

#### 5.5.3. Attention Mechanism

The attention mechanism is an important mechanism pioneered in the field of nlp and has also been developed in object detection in recent years. In order to verify the effect of adding an attention mechanism in different layers of ResNet101, we conducted a sensitivity analysis for the number of times an attention mechanism is introduced while retaining the other conditions. As shown in Table 7, when we added the attention mechanism to the first three layers of ResNet101, the detection effect improved to a certain extent. However, continuing to introduce attention-blocks containing edge information to the 4th and 5th layers will cause a drop in detection accuracy. This is because there is more abstract information in the feature maps extracted by the 4th and 5th layers in ResNet101, and the edge information is the basic feature information. This is counterproductive and reduces the detection performance.

#### 5.5.4. Number of Epochs

The number of epochs for experimental training will affect the performance of the model. If the number of training epochs is not enough, the model will be under-fitted, and the model will not yet have fully learned to identify all the objects to be detected. Excessive training epochs will reduce the robustness of the model, the parameters will be limited by the existing training data, and the realization of unfamiliar data in the test set will be reduced. Therefore, we conducted an evaluation test of the number of training times for the performance of the model, and the test results are shown in Table 8. It can be seen from the table that when the training epoch number was 200, the model was the most balanced.

#### 5.5.5. Minimum Training Data Experiment

Changes in the amount of training data will also affect the final performance of the model. At the same time, by comparing the detection accuracy of the model under different amounts of data, we can judge the feature extraction ability of the model. As shown in Table 9, we conducted experiments with the minimum amount of data. From the results, it can be seen that, when the amount of data decreased, the performance of the model also had weak performance, which indicates that the amount of our data was sufficient. The model performance did not drop significantly until the test set dropped to 1750. Moreover, INSU-YOLO has strong robustness and can still learn key feature information on small-scale datasets, which overcomes the shortcomings of the previous model’s poor generalization ability to a certain extent.

### 5.6. Ablation Analysis

To analyze the functions of the different components of INSU-YOLO, we performed an ablative analysis on InsuDetSet. As shown in Table 10, Model B had better indicators than Model A, which indicates that using ResNet101 as the backbone can better extract image features. Model C uses image augment for preprocessing, which improves the quality of the input image and provides the model with better training data. Compared with other stages, the performance of Model D showed the highest improvement in detection effect. This indicates that the attention mechanism plays a sufficient role, because the attention mechanism allows the model to focus on the edge information of the insulator when converging, with the help of the image enhancement model. In addition, it can be seen from other comparative experiments that the additional overhead brought by it is very low, so it is necessary for our task to add an attention mechanism to the backbone.

### 5.7. Computational Complexity

The network parameters and training time were recorded to evaluate the space and time complexity of the networks. As shown in Table 11, compared with Cascade R-CNN, INSU-YOLO has a similar detection effect, but its parameters and training time are greatly reduced. Compared with YOLOv4, the space complexity and the training time are basically unchanged, because we only changed the backbone and added the attention mechanism on its basis, but a higher detection effect was achieved. In addition, CenterNet still consumes the least resources. The computational complexity of SSD is slightly higher than that of RetinaNet, but the detection effect is slightly worse.

## 6. Conclusions

This paper proposes a missing-sheds granularity estimation of glass insulators based on deep neural networks, which can obtain insulator identification and defect degree estimation results based on a real insulator set. The model first uses an image augment module to improve the quality of the input image and to provide edge images, and then uses an INSU-YOLO framework to obtain detection frames for insulators and defects. The degree of defects is finally obtained based on missing-sheds granularity estimation. Quantitative and qualitative experiments on the self-built InsuDetSet dataset provided the following results: Compared with current mainstream models, the INSU-YOLO proposed in this paper can output accurate insulator-detection images. The sensitivity analysis experiment demonstrated that the attention mechanism introduced in this paper significantly improved the ability to detect insulators and defects. Missing-sheds granularity estimation yields a superior assessment of the threat level of defects. In addition, we have only performed defect detection on glass insulators on high-voltage overhead power lines, and we believe that our model is equally applicable to ceramic and composite insulators and other electrical components. Therefore, we will continue to improve the detection accuracy of the proposed method at the next stage and conduct experiments on other power components when the amount of data is sufficient.

## Figures and Tables

**Table 1:** Applied kernels of ResNet101 in INSU-YOLO.

Layer	Output Size	Kernel Size
conv1	304 × 304	7 × 7, 64
conv2_x	152 × 152	$\left[\begin{array}{c} 1\times 1,64\\ 3\times 3,64\\ 1\times 1,\;256 \end{array}\right]$× 3
conv3_x	76 × 76	$\left[\begin{array}{c} 1\times 1,128\\ 3\times 3,128\\ 1\times 1,512 \end{array}\right]$× 4
conv4_x	38 × 38	$\left[\begin{array}{c} 1\times 1,256\\ 3\times 3,256\\ 1\times 1,1024 \end{array}\right]$× 23
conv5_x	19 × 19	$\left[\begin{array}{c} 1\times 1,512\\ 3\times 3,512\\ 1\times 1,2048 \end{array}\right]$× 3

**Table 2:** Applied kernels of ResNet101 in INSU-YOLO.

Defect Granularity Level	Ratio
Normal	0
Fine	(0, 0.02]
Large	(0.02, 1]

**Table 3:** APs of the different models.

Model	InsuDetSet			FPS
	AP_50_	AP_70_	AP_90_	
YOLOv4	89.14	81.58	74.37	72
SSD	86.82	79.17	72.29	70
RetinaNet	88.29	80.73	73.81	74
CenterNet	85.38	78.34	70.53	117
Cascade R-CNN	92.26	89.52	80.34	32
INSU-YOLO	93.73	90.58	82.15	76

**Table 4:** Accuracy of defect detection.

Model	Normal	Fine	Large	Neglected
YOLOv4	0.52	0.33	0.13	0.02
SSD	0.48	0.38	0.11	0.03
RetinaNet	0.48	0.38	0.12	0.02
CenterNet	0.47	0.34	0.12	0.07
Cascade R-CNN	0.49	0.34	0.16	0.01
INSU-YOLO	0.50	0.34	0.15	0.01
Ground Truth	0.50	0.36	0.14	0

**Table 5:** APs of different backbones.

Backbone	InsuDetSet			FPS
	AP_50_	AP_70_	AP_90_	
CSPDarknet53	89.14	81.58	74.37	72
ResNet50	85.03	78.72	70.64	79
ResNet101(ours)	93.73	90.58	82.15	76
ResNet152	94.16	90.92	84.23	64

**Table 6:** APs of different preprocessing methods.

Preprocessing Method	InsuDetSet			FPS
	AP_50_	AP_70_	AP_90_	
None preprocessing	88.27	80.63	72.94	80
Image denoising	89.03	82.84	74.76	79
Edge extraction	91.46	85.63	78.28	78
Image denoising + edge extraction	93.73	90.58	82.15	76

**Table 7:** APs of different introduction times of the attention mechanism.

Introduced Layer	InsuDetSet			FPS
	AP_50_	AP_70_	AP_90_	
None	87.39	78.45	70.06	82
C1	88.94	81.54	72.75	80
C1, C2	90.57	85.72	78.39	78
C1, C2, C3	93.73	90.58	82.15	76
C1, C2, C3, C4	93.25	89.84	81.63	75
C1, C2, C3, C4, C5	89.16	83.32	74.27	73

**Table 8:** APs of different epoch numbers.

Number of Epochs	InsuDetSet			FPS
	AP_50_	AP_70_	AP_90_	
50	72.49	61.50	42.27	80
100	81.62	73.38	66.14	78
150	85.24	81.07	75.49	76
200	93.73	90.58	82.15	76
250	93.28	88.73	79.26	75

**Table 9:** Results of minimum training data experiment.

The Amount of Training Set	InsuDetSet			FPS
	AP_50_	AP_70_	AP_90_	
2500 (100%)	93.73	90.58	82.15	76
2250 (90%)	90.62	87.37	79.94	75
2000 (80%)	86.40	82.86	76.32	74
1750 (70%)	83.52	78.21	72.59	74
1500 (60%)	74.08	70.73	65.01	72

**Table 10:** The results of the ablation analysis.

Model	Architecture	AP_50_	AP_70_	AP_90_
A	YOLOv4	87.39	79.54	71.82
B	A + ResNet101	89.57	83.26	74.91
C	B + image augment	90.93	85.45	77.60
D	C + attention mechanism	93.73	90.58	82.15

**Table 11:** Network parameters (Param.) and training time of the different models.

Model	Param.	Training Time (h)
YOLOv4	28 M	6.38
SSD	34 M	7.46
RetinaNet	32 M	7.03
CenterNet	14 M	4.05
Cascade R-CNN	184 M	49.84
INSU-YOLO	30 M	6.92

## Data Availability

The data in this paper are undisclosed due to the confidentiality requirements of the data supplier.
